# Dynamic Response of Tapered Optical Multimode Fiber Coated with Carbon Nanotubes for Ethanol Sensing Application

**DOI:** 10.3390/s150510452

**Published:** 2015-05-04

**Authors:** Arafat Shabaneh, Saad Girei, Punitha Arasu, Mohd Mahdi, Suraya Rashid, Suriati Paiman, Mohd Yaacob

**Affiliations:** 1 Wireless and Photonics Network Research Centre, Engineering and Technology Complex, Universiti Putra Malaysia, 43400 UPM Serdang, Selangor, Malaysia; E-Mails: tamimi_127@hotmail.com (A.S.); gireisaad3@gmail.com (S.G.); punitha_arasu@yahoo.com (P.A.); mam@upm.edu.my (M.M.); 2 Photonics Laboratory, Department of Computer & Communications Systems Engineering, Faculty of Engineering, Universiti Putra Malaysia, 43400 UPM Serdang, Selangor, Malaysia; 3 Department of Chemical and Environmental Engineering, Faculty of Engineering, Universiti Putra Malaysia, 43300 Serdang, Selangor, Malaysia; E-Mail: suraya_ar@upm.edu.my; 4 Department of Physics, Faculty of Science, Universiti Putra Malaysia, 43400 Serdang, Selangor, Malaysia; E-Mail: suriati@upm.edu.my

**Keywords:** carbon nanotubes, tapered optical fiber, ethanol sensing, reflectance

## Abstract

Ethanol is a highly combustible chemical universally designed for biomedical applications. In this paper, optical sensing performance of tapered multimode fiber tip coated with carbon nanotube (CNT) thin film towards aqueous ethanol with different concentrations is investigated. The tapered optical multimode fiber tip is coated with CNT using drop-casting technique and is annealed at 70 °C to enhance the binding of the nanomaterial to the silica fiber tip. The optical fiber tip and the CNT sensing layer are micro-characterized using FESEM and Raman spectroscopy techniques. When the developed sensor was exposed to different concentrations of ethanol (5% to 80%), the sensor reflectance reduced proportionally. The developed sensors showed high sensitivity, repeatability and fast responses (<55 s) towards ethanol.

## 1. Introduction

Carbon nanotubes (CNT) have attracted considerable attention the last decade since its discovery in 1991 [1,2,3,4,5], due to their unique structure and properties. CNT has many features such as high mechanical strength and chemical stability as well as good optical and electrical properties [1,6,7,8,9,10,11,12]. CNT are also extensively used for the improvement of electrochemical sensors [13]. 

There are several interesting works reporting on optical fiber based chemical sensors. Zheng *et al.* [14] reported a nano-film coated photonic crystal fiber (PCF) long-period grating (LPG) based sensors for moisture detection. They had fabricated two types of sensors coated with interior and exterior nano-films. Their work showed that the sensors coated with interior nano-films have high resonance wavelength shift of 0.0007%/pm and provide a good platform for moisture detection [14]. The fast development in the applications of optical components as result of immense commercialization in optoelectronic and telecommunication has shifted the focus of researchers and manufacturers to its application in optical sensing, which is new and less explored compared to electrical sensors. Owing to the properties of optical signals, its benefits are numerous compared to electrical signals. Among the significance of optical signals are: immunity to electromagnetic interference, resistance to corrosion and safe to deploy in flammable environments giving rise to applications of optical sensors in chemical sensing [15]. It has been reported that sensitivity increased by integrating nanomaterials on the sensing area of fiber optic sensors [16].

Tapered optical fiber is one of the most suitable optical transducing platforms for sensing applications. Tapered optical fiber is more sensitive as compared to the conventional fiber due to the behavior of light propagation in its core. A larger fraction of the optical power propagates outside the tapered fiber core as compared to the normal fiber core and therefore, allowing the interaction of the light with the sensing layer [17]. However, the integration of CNT onto tapered optical multi-mode fiber (MMF) as a chemical sensor is yet to be fully investigated. It is expected that CNT with its unique properties when integrated with tapered fiber will lead to the improvement in the device sensitivity [18,19].

Ethanol is a popular psychoactive liquid which is taken by people especially in rural areas which intoxicate them [20]. It is best known as alcohol from alcoholic beverages; it also serves as thermometric liquid, and as a fuel [21]. It burns without smoke with blue flame that might not visible in normal light [22]. Ethanol is widely used in medical as well as chemical industries. Therefore, the monitoring of ethanol concentration in other liquids is of paramount importance. Moreover, there is need to detect and monitor ethanol with the aid of optical sensors that are reliable to prevent hazards as ethanol is a flammable as well as corrosive liquid.

One of the advantages of optical sensors is its room temperature operation thus reducing power consumption and complex circuitry. Therefore, there are exciting opportunities in the investigation of the sensing properties of CNT nanostructured thin film deposited onto tapered optical fiber tip. This manuscript presents performance analysis of ethanol sensor based on tapered MMF tip coated with CNT via drop coating technique. The micro characterization result of the CNT thin film is investigated via field emission scanning electron microscopy (FESEM) and Raman spectroscopy techniques. The dynamic responses of the developed fiber sensor with CNT towards different concentrations of ethanol (5% to 80%) are also reported.

## 2. Experimental Details

### 2.1. Tapered Optical Fiber 

**Figure 1:**
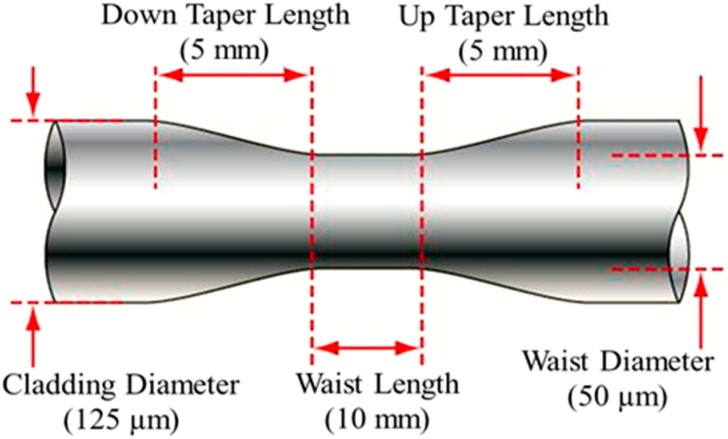
Tapered fiber parameters.

A standard optical MMF with core and cladding diameter of 62.5 µm and 125 µm respectively is deployed to fabricate the tapered fiber tip. Tapered fiber is fabricated using VYTRAN glass processing workstation (GPX 3400 series, Morganville, NJ, USA). This machine operates by pulling the two ends of the fiber while it is heated by the filament. The polymeric coating of the bare optical fiber is removed mechanically and followed by chemical cleaning using alcohol. The fiber is placed on the tapering machine by a fiber holding block (FHB), which controls the pulling distance from both ends. The area to be tapered is positioned above the filament clamp. Taper parameters are determined utilizing proprietary operating software. The transitions and the waist lengths are set to 5 mm and 10 mm, respectively. The waist diameter is set to 50 µm as illustrated in Figure 1. Later, the fiber is cleaved using a cleaver in the center of the tapered region to achieve waist length tip of 5 mm.

### 2.2. Deposition of CNT Nanostructured Thin Films

To achieve functionalized CNT which can be simply dispersed in acetone, the CNT powder (from Hangzhou Company, China) is soaked in nitric acid (HNO_3_) as a treatment procedure [23]. Then, the CNT powder is dispersed in acetone by ultrasonication method. The concentration of CNT utilized in this experiment is 0.0025 g/mL. CNT thin film is deposited on silica (SiO_2_) substrates via drop casting technique for characterization purposes and on the tapered optical MMF tip for ethanol sensing investigation. Prior to the CNT deposition, the SiO_2_ wafer substrate and the fiber tip are cleaned with alcohol and heated in an oven at 70 °C for 10 min to enhance the binding of the nanomaterial. After that, the samples are heated up again in the oven for 10 min at 70 °C after drop casting the CNT, and left to dry in the air to improve the film adhesion.

The micro-characterization results of the CNT thin film in this experiment are investigated using field emission scanning electron microscopy (FESEM) and Raman spectroscopy. The FESEM image is also processed employing an ImageJ^®^ (image processing software) to extract the average diameter of the CNT tubes.

### 2.3. Experimental Setup

**Figure 2:**
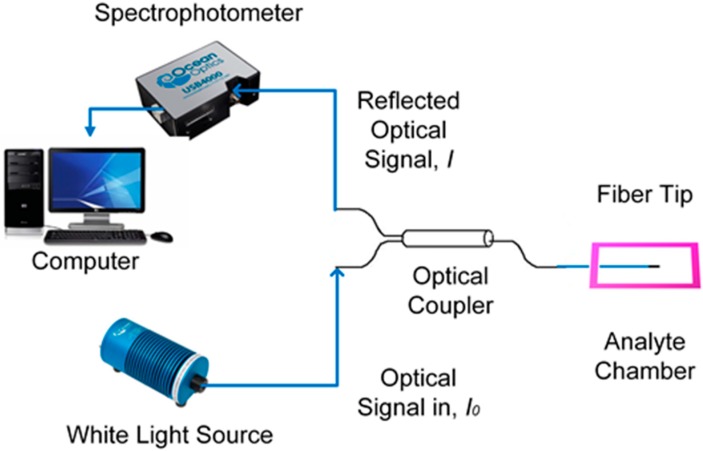
Experimental setup.

The experimental setup consists of a white light source (HL-2000, Ocean Optics, Dunedin, FL, USA) and a spectrophotometer (USB4000, Ocean Optics, Dunedin, FL, USA) as shown in Figure 2. The tapered optical MMF tip coated with CNT thin film is connected to a multi-mode coupler (2 × 1 multimode optical coupler). The coupler is connected to the light source and the spectrophotometer using optical cable connecters. The reflected light from the optical MMF tip passes through the coupler and is collected by the spectrophotometer. Following this, the data from the sensor is processed by the computer via SpectraSuite software.

## 3. Results and Discussion

### 3.1. Tapered Optical Fiber Properties

**Figure 3:**
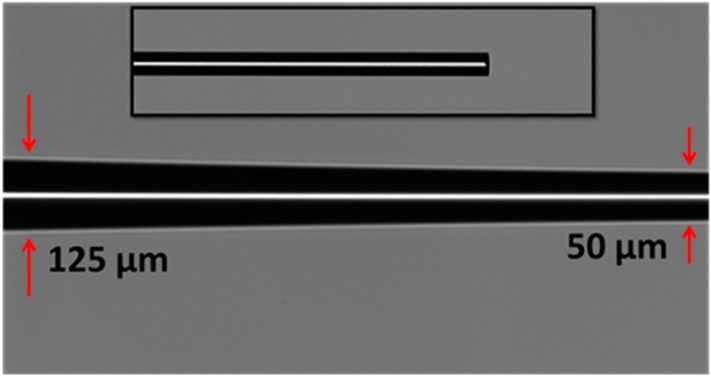
The image of the fabricated tapered optical fiber. The inset shows the tip of the tapered optical fiber.

Figure 3 illustrates the image of the fabricated tapered optical MMF captured by the glass workstation CCD camera. The waist diameter of the fiber reduces linearly from 125 to 50 µm. The inset shows the tip of the tapered optical MMF after cleaving. The tapered fiber can be considered adiabatic if the main portion of the power remains in the fundamental mode (LP_01_) and does not couple to higher order modes as it propagates along the taper [24,25]. Tapered fiber transition region with adiabatic criterion, as shown in Figure 3, has sufficiently lengthened the transition region with very small change in taper’s radius (small taper angle). Hence, the loss of propagating power through the optical fiber as a result of tapering is decreased. The loss of the propagating signal after optical fiber tapering is measured to be 1.1 dB.

### 3.2. CNT Film Characterizations

**Figure 4:**
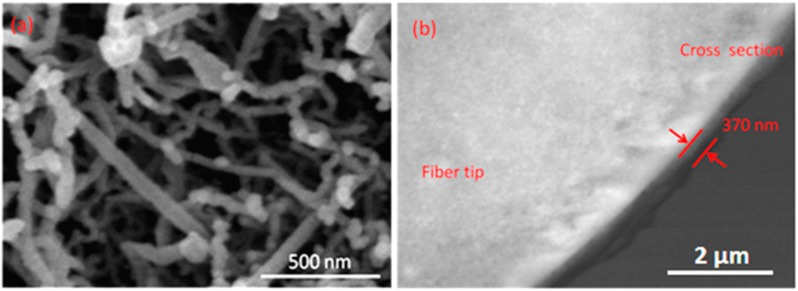
Field emission scanning electron microscopy (FESEM) images of carbon nanotube (CNT) (**a**) deposited on SiO_2_ substrate (**b**) Cross-section deposited on tapered optical fiber tip.

The morphology of the CNT thin film layers is observed using FESEM. The images are obtained using FESEM S-3400N Hitachi. Figure 4a shows the image of CNT deposited on SiO_2_ substrate. A densely entangled bundled structure of MWCNTs can be seen clearly using an accelerating voltage of 20.0 kV source. Moreover, the CNT are organized in bundles with individual nanotubes arranged in a dense net with an excellent adhesion to the substrate as previously reported [26]. The FESEM image is also processed employing an ImageJ^®^ (image processing software) to extract the average diameter of the tubes, which was determined to be approximately ~35 nm. Figure 4b shows the cross-section FESEM image of the tapered optical fiber tip coated with CNT. The thickness of the CNT on the fiber tip is measured to be 370 nm. This FESEM is performed to assure uniform coating of CNT films on the substrates.

**Figure 5:**
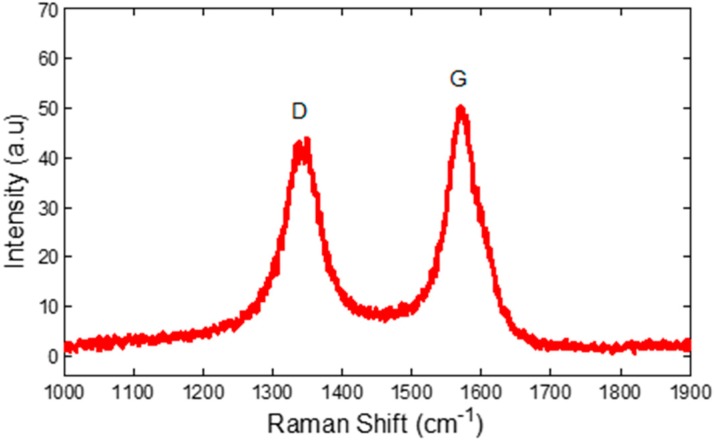
Raman shift of the CNT nanostructured thin films.

Raman spectroscopy with laser wavelength of 514 nm (Horiba Jobin Yvon, LabRam HR800) is utilized for the characterizations of the CNT nanostructured thin film. Figure 5 shows the Raman spectrum of the CNT on glass substrates. The spectrum highlights two sharp peaks that are unique to the CNT. The D-band position at 1355 cm^−1^ is a result from the disorder occurred in the CNTs. Meanwhile, the G-band position at 1590 cm^−1^ corresponds to graphitic and well-ordered carbon atoms [27,28]. One of the most important properties in Raman spectra for CNT is the tangential stretch of G-band. The I_d_/I_g_ ratio is 0.9 which indicates highly graphitized and less defective carbon nanotubes [28]. These microcharacterization results are important to identify the morphology of the CNT nanostructured thin films.

### 3.3. Optical Reflectance Spectra Performance

**Figure 6:**
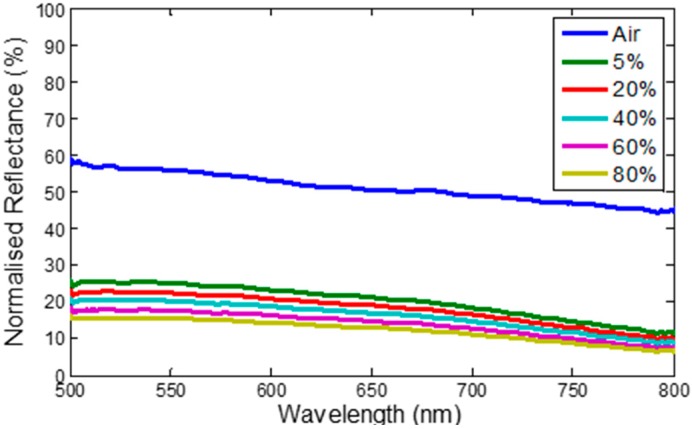
Reflectance spectra of sensor with different ethanol concentrations in distilled water.

Figure 6 shows the normalised reflection spectra of tapered optical MMF tip coated with CNT exposed to air and different concentrations of ethanol at room temperature. The reflectance is normalised to the air as a reference. It has been observed that the reflectance spectrum of the CNT coated fiber tip decreases when exposed to 5%, 20%, 40%, 60% and 80% concentrations of ethanol through a wavelength range of 500–800 nm as shown in Figure 6. In a tapered fiber, a higher fraction of evanescent wave field passes inside the cladding, which is sensitive to the physical environment of its’ surroundings [19,29]. The interaction between the CNT on the tapered fiber tip and different concentrations of ethanol [30] molecules transforms the optical properties of the CNT film, resulting in the response of the developed sensors towards ethanol. 

### 3.4. Ethanol Sensing Response

**Figure 7:**
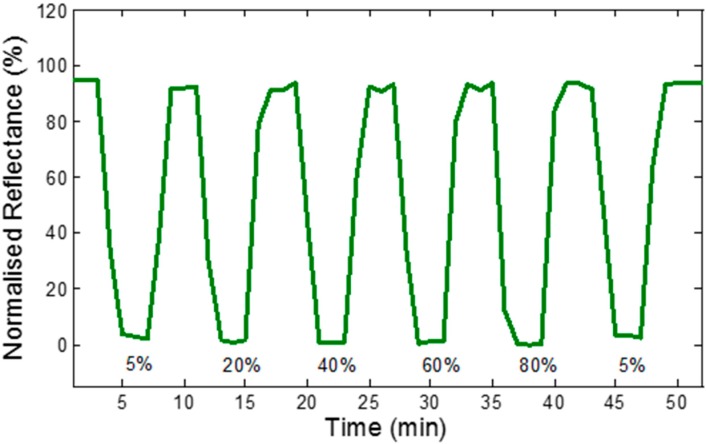
Dynamic response of uncoated sensor, towards different concentrations of ethanol, integrated over wavelength range of 500–800 nm.

**Figure 8:**
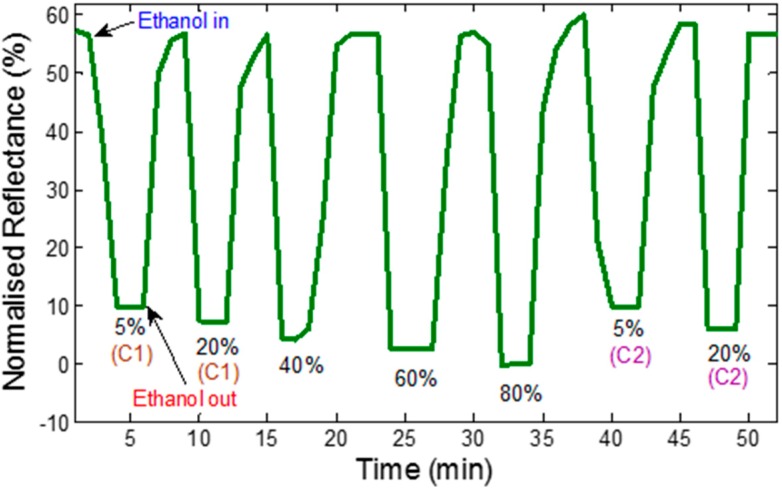
Dynamic response of the CNT thin film to different concentrations of ethanol operating at room temperature, integrated over wavelength range of 500–800 nm.

Figure 7 and Figure 8 demonstrate the presence of ethanol which is detected by fiber tips without and with CNT thin films, respectively. The experiments are conducted at room temperature. Figure 7 displays the dynamic response of blank optical tapered fiber tip exposed towards different concentrations of ethanol in distilled water. The measurement is taken by integrating the spectrum over a wavelength range of 500–800 nm. It indicates high sensitivity of the optical fiber tip towards ethanol. Nevertheless, the responses of the blank fiber tip did not distinguish the different ethanol concentrations, and these are expected to be only due to the change of the ethanol refractive index.

In contrast, the dynamic response is proportional to the different concentrations of ethanol solutions exposed to the tapered optical MMF tip coated with CNT nanostructured thin film as shown in Figure 8. The response from the CNT coated fiber tip changes according to the ethanol concentrations in the forward order. This is believed to be due to the change of refractive index as well as chemisorption of ethanol onto CNT layer.

**Figure 9:**
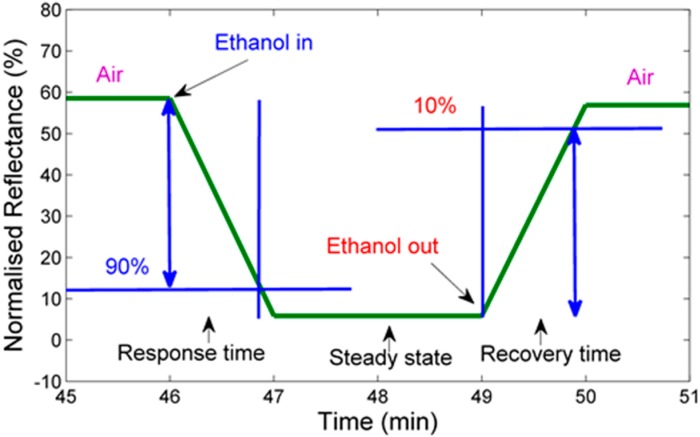
Dynamic response curve showing response and recovery time estimation.

A significant decrease in the reflectance is observed with respect to the response time [31]. However, the reflectance response time is descending with the increase in the concentrations of ethanol. The dynamic reflectance response decreased by 58% when the sensor is exposed to 80% of ethanol. The sensor recovered fully and returned to its initial base line upon exposure to air. The response and recovery times of the sensor are calculated based on the response in Figure 8. The developed sensor shows fast as well as stable response and recovery. Based on the definition of response time as the duration it takes to rise 90% of the total magnitude, and recovery time as the duration it takes to recover to 10% of its baseline, the response and recovery times are determined as shown in Figure 9 [32]. In general, the response time for the CNT coated fiber tip is 50 s, while the recovery time is 53 s. The developed sensor shows high sensitivity as well as fast responses and recovery as compared to Penza *et al.* [5] and Someya *et al.* [33] works. Furthermore, the sensor shows stable responses, repeatability and reversibility. The integrated wavelength over the range of 500–800 nm measurement method was introduced to optimize the quantification of the response and hence improve the sensors’ sensitivity towards low ethanol concentrations. This measurement technique was introduced in the previous publication [34]. 

Repeatability is the ability of a sensor to produce the same response when the tests are repeated multiple times. The parameter is important to demonstrate the reliability of the sensors. The sensors show high repeatability by having similar reflectance response towards a couple of ethanol cycles especially with 5% and 20% concentrations as shown also in Figure 8. It can be noted that the reflectance dip for the first ethanol cycle with 5% (C1) and 20% (C1) concentrations is 10% and 8%, respectively. The response magnitude was demonstrated to be the same in the second cycle of the experiment as shown by the reflectance of 5% (C2) and 20% (C2) concentrations. The result reveals a promising performance of CNT coated optical MMF tip as a highly sensitive ethanol sensor.

**Figure 10:**
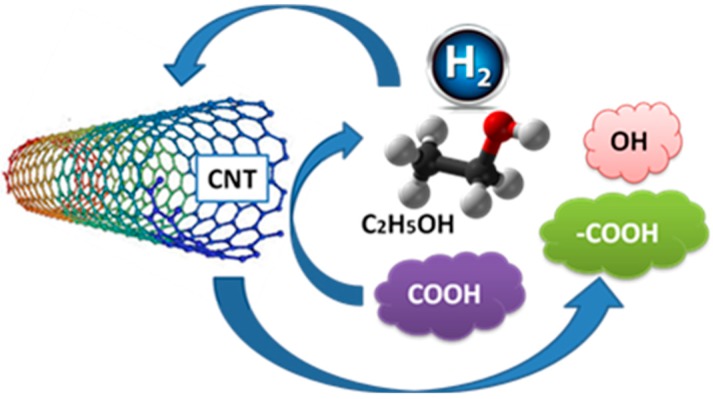
Schematic representation of ethanol sensing at CNT surface.

The sensing mechanism of ethanol was suggested by Ouyang [35] and Shabaneh [20]. Figure 10 shows an illustration of ethanol sensing at CNT surface. It is proposed that a stronger response is shown towards ethanol when the polar COOH groups attached onto the nanotube surface. The absorption efficiency of ethanol molecules will improve owing to the reality that there are hydrogen bonding of dipole-dipole interactions between the COOH groups on the CNT and the polar organic molecules of ethanol. The –COOH group and the OH group of ethanol molecules in the liquid chamber attach to the CNT nanostructured thin film and will interact through hydrogen bonds. This leads to dynamic change of the ethanol sensor output [35]. Finally, the O–H stretch of the water molecules is decreased due to the presence of carbon molecules from ethanol.

**Figure 11:**
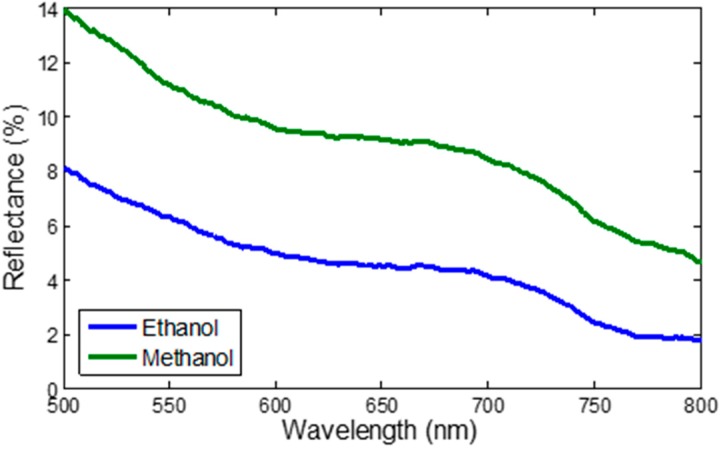
Reflectance response of tapered fiber coated with CNT towards pure methanol and ethanol.

The sensor was tested with methanol to investigate its’ performance towards other volatile organic compounds (VOCs). Figure 11 compares the reflectance response of the sensor for 100% ethanol and methanol. It can be observed that the reflectance is higher when the sensor is exposed to methanol than when it is exposed to ethanol. This is due to the difference in the chemicals’ refractive indices (methanol = 1.3326 and ethanol = 1.3627). The difference in the reflectance magnitude indicates a degree of the selectivity of the sensor towards different VOCs. 

**Figure 12:**
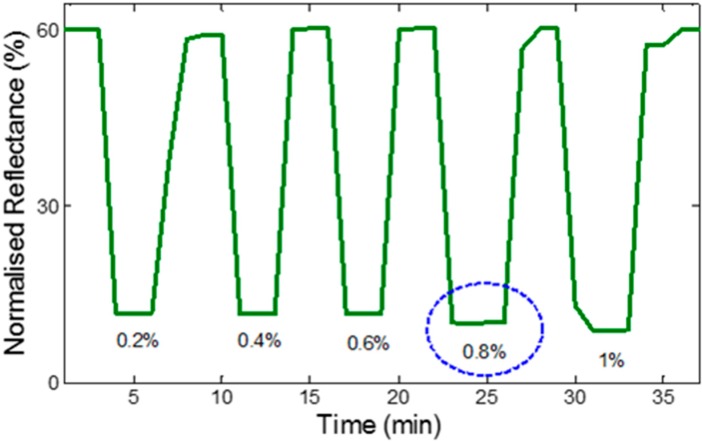
Resolution of the developed fiber sensor.

Resolution signifies the smallest incremental change in the measurand that will result in a detectable increment in the output signal. Resolution is strongly limited by any noise in the signal. Figure 12 shows the resolution of the developed fiber sensor with low ethanol concentrations. The sensor demonstrated resolution limit of 0.8% concentration of ethanol over a wavelength range of 500–800 nm as shown in Figure 12.

### 3.5. Sensitivity and Linearity of the Ethanol Fiber Sensors

**Figure 13:**
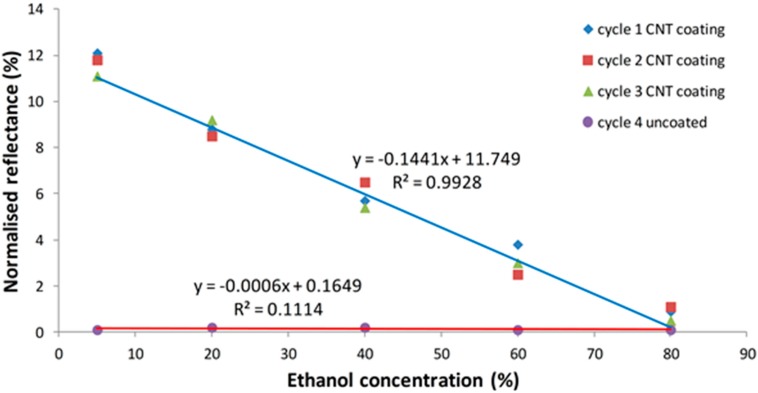
Reflectance changes against ethanol concentrations of blank tapered and CNT coated tapered fiber.

It can be observed that the CNT coated on tapered optical fiber sensor shows higher sensitivity than the uncoated sensor. Figure 13 shows the calibration curve for the CNT coated sensor and the blank tapered optical fiber tip. The sensitivity of the CNT coated fiber sensor is 0.1441/vol% ethanol concentration and has slope linearity of more than 99% (cycle 1–3). While the sensitivity of the uncoated and tapered optical fiber is shown to be 0.0006/vol% ethanol concentration and linearity of 11% (cycle 4). At the same time, the sensitivity stability tests have been performed as shown in Figure 13. Overall, results show that the interactions of different ethanol concentrations with CNT thin films make the sensor highly sensitive when compared to uncoated and tapered fiber.

## 4. Conclusions

A tapered optical multi-mode fiber tip coated with CNT nanostructured thin film towards aqueous ethanol with different concentration in distilled water is successfully developed and investigated. CNT nanostructured thin film as the sensing layer is produced via drop casting method onto the fiber tip. The sensor reflectance demonstrates a linear correlation with ethanol concentration in range of 5% to 80% in distilled water. The developed sensor shows high sensitivity, fast and stable responses. The sensor is characterized by an excellent dynamic performance with both response and recovery time of 50 s and 53 s respectively, in the visible spectrum range at room temperature. As a result, the ethanol sensing performance of the developed sensors point out their potential in food analysis and medical sensing usages.

## References

[1] Brahim S., Colbern S., Gump R., Moser A., Grigorian L. (2009). Carbon nanotube-based ethanol sensors. Nanotechnology.

[2] Consales M., Crescitelli A., Penza M., Aversa P., Veneri P.D., Giordano M., Cusano A. (2009). SWCNT nano-composite optical sensors for VOC and gas trace detection. Sens. Actuators B Chem..

[3] Mahdavian L., Monajjemi M., Mangkorntong N. (2009). Sensor response to alcohol and chemical mechanism of carbon nanotube gas sensors. Fuller. Nanotub. Carbon Nanostruct..

[4] Moshkalyov S., Moreau A., Guttiérrez H., Cotta M., Swart J. (2004). Carbon nanotubes growth by chemical vapor deposition using thin film nickel catalyst. Mater. Sci. Eng. B.

[5] Penza M., Cassano G., Aversa P., Antolini F., Cusano A., Cutolo A., Giordano M., Nicolais L. (2004). Alcohol detection using carbon nanotubes acoustic and optical sensors. Appl. Phys. Lett..

[6] Girei S., Shabaneh A., Arasu P., Painam S., Yaacob M. Tapered multimode fiber sensor for ethanol sensing application. Proceedings of the 2013 IEEE 4th International Conference Photonics (ICP).

[7] Jang J., Bae J. (2007). Carbon nanofiber/polypyrrole nanocable as toxic gas sensor. Sens. Actuators B Chem..

[8] Lin Y., Yantasee W., Wang J. (2005). Carbon nanotubes (CNTs) for the development of electrochemical biosensors. Front. Biosci..

[9] Liu S., Cai C. (2007). Immobilization and characterization of alcohol dehydrogenase on single-walled carbon nanotubes and its application in sensing ethanol. J. Electroanal. Chem..

[10] Nguyen L.Q., Phan P.Q., Duong H.N., Nguyen C.D., Nguyen L.H. (2013). Enhancement of NH_3_ gas sensitivity at room temperature by carbon nanotube-based sensor coated with Co nanoparticles. Sensors.

[11] Slepyan G.Y., Shuba M., Maksimenko S., Lakhtakia A. (2006). Theory of optical scattering by achiral carbon nanotubes and their potential as optical nanoantennas. Phys. Rev. B.

[12] Kashiwagi K., Yamashita S., Set S.Y. (2009). *In-situ* monitoring of optical deposition of carbon nanotubes onto fiber end. Opt. Express.

[13] Hu C., Hu S. (2009). Carbon nanotube-based electrochemical sensors: Principles and applications in biomedical systems. J. Sens..

[14] Zheng S., Zhu Y., Krishnaswamy S. (2013). Fiber humidity sensors with high sensitivity and selectivity based on interior nanofilm-coated photonic crystal fiber long-period gratings. Sens. Actuators B Chem..

[15] Ritchie R.J. (2006). Consistent sets of spectrophotometric chlorophyll equations for acetone, methanol and ethanol solvents. Photosynth. Res..

[16] Sekimoto S., Nakagawa H., Okazaki S., Fukuda K., Asakura S., Shigemori T., Takahashi S. (2000). A fiber-optic evanescent-wave hydrogen gas sensor using palladium-supported tungsten oxide. Sens. Actuators B Chem..

[17] Guo S., Albin S. (2003). Transmission property and evanescent wave absorption of cladded multimode fiber tapers. Opt. Express.

[18] Kieu K.Q., Mansuripur M. (2006). Biconical fiber taper sensors. IEEE Photon. Technol. Lett..

[19] Shabaneh A., Girei S., Arasu P., Rahman W., Bakar A., Sadek A., Lim H.N., Huang N.M., Yaacob M.H. (2014). Reflectance response of tapered optical fiber coated with graphene oxide nanostructured thin film for aqueous ethanol sensing. Opt. Commun..

[20] Shabaneh A., Girei S., Arasu P., Rashid S., Yunusa Z., Mahdi M., Paiman S., Ahmad M.Z., Yaacob M.H. (2014). Reflectance Response of Optical Fiber Coated with Carbon Nanotubes for Aqueous Ethanol Sensing. IEEE Photon. J..

[21] Sun Z., Zhang X., Na N., Liu Z., Han B., An G. (2006). Synthesis of ZrO_2_-carbon nanotube composites and their application as chemiluminescent sensor material for ethanol. J. Phys. Chem. B.

[22] Valentini L., Cantalini C., Armentano I., Kenny J., Lozzi L., Santucci S. (2004). Highly sensitive and selective sensors based on carbon nanotubes thin films for molecular detection. Diam. Relat. Mater..

[23] Wei B.Y., Hsu M.C., Su P.G., Lin H.M., Wu R.J., Lai H.J. (2004). A novel SnO_2_ gas sensor doped with carbon nanotubes operating at room temperature. Sens. Actuators B Chem..

[24] Leung A., Shankar P.M., Mutharasan R. (2007). A review of fiber-optic biosensors. Sens. Actuators B Chem..

[25] Wu R.J., Huang Y.C., Yu M.R., Lin T.H., Hung S.L. (2008). Application of m-CNTs/NaClO_4_/Ppy to a fast response, room working temperature ethanol sensor. Sens. Actuators B Chem..

[26] Zibaii M., Latifi H., Karami M., Gholami M., Hosseini S., Ghezelayagh M. (2010). Non-adiabatic tapered optical fiber sensor for measuring the interaction between α-amino acids in aqueous carbohydrate solution. Meas. Sci. Technol..

[27] DiLeo R.A., Landi B.J., Raffaelle R.P. (2007). Purity assessment of multiwalled carbon nanotubes by Raman spectroscopy. J. Appl. Phys..

[28] Bokobza L., Zhang J. (2012). Raman spectroscopic characterization of multiwall carbon nanotubes and of composites. Express Polym. Lett..

[29] Xiong F., Zhu W., Lin H., Meng X. (2014). Fiber-optic sensor based on evanescent wave absorbance around 2.7 μm for determining water content in polar organic solvents. Appl. Phys. B.

[30] Arasu P., Noor A., Shabaneh A., Girei S., Mahdi M., Lim H., Rashid H.A., Yaacob M.H. (2014). Absorbance properties of gold coated fiber Bragg grating sensor for aqueous ethanol. J. Eur. Opt. Soc-Rapid.

[31] Shabaneh A., Arasu P., Girei S., Paiman S., Mahdi M., Huang N.M., Yaacob M. Reflectance response of optical fiber sensor coated with graphene oxide towards ethanol. Proceedings of the 2013 IEEE 4th International Conference on Photonics (ICP).

[32] Cao W., Duan Y. (2005). Optical fiber-based evanescent ammonia sensor. Sens. Actuators B Chem..

[33] Someya T., Small J., Kim P., Nuckolls C., Yardley J.T. (2003). Alcohol vapor sensors based on single-walled carbon nanotube field effect transistors. Nano Lett..

[34] Ou J., Yaacob M., Campbell J., Kalantar-Zadeh K., Wlodarski W. (2012). H_2_ sensing performance of optical fiber coated with nano-platelet WO_3_ film. Sens. Actuators B Chem..

[35] Ouyang M., Li W.J. Reusable CNTs-based chemical sensors. Proceedings of the 2009 IEEE International Conference on Nano/Molecular Medicine and Engineering (NANOMED).

